# The Evolution of Lupus Nephritis Therapy from the 1960s to the Present

**DOI:** 10.3390/bioengineering13040428

**Published:** 2026-04-06

**Authors:** Wenjuan Zhu, Haiyan He, Xiaoyan Huang, Lijun Zhang, Pearl Pai

**Affiliations:** 1 Division of Nephrology, Department of Medicine, University of Hong Kong-Shenzhen Hospital, Shenzhen 518053, China; 2 Shenzhen Clinical Research Center for Rare Diseases, Shenzhen 518053, China; huangxy@hku-szh.org; 3 Division of Rheumatology, Department of Medicine, University of Hong Kong-Shenzhen Hospital, Shenzhen 518053, China; 4 Department of Medicine, LKS Faculty of Medicine, University of Hong Kong, Pokfulum, Hong Kong SAR, China

**Keywords:** lupus nephritis, systemic lupus erythematosus, immunosuppressive therapy, belimumab, CAR-T cell therapy

## Abstract

Lupus nephritis (LN) stands out as one of the most critical complications of systemic lupus erythematosus (SLE), affecting almost 60% of the patient population. Even though more therapies have been made available for LN in the past decade, clinical outcomes remain less than ideal: nearly 10% to 30% of LN cases still advance to end-stage kidney disease (ESKD), still making the management of LN a clinical challenge. Therefore, the primary aim of treatment of LN is simple: to halt the progression toward chronic kidney disease (CKD) and prevent renal failure. In this review, we briefly describe the immunopathological basis of LN, which provides the scientific rationale for new drug development. We will focus on the current in-use medications, especially on proliferative LN, building on the legacy of the 20th century, and we will outline new emerging targeted and innovative therapies. We will also present the standard-of-care as informed by international guidelines and review the management of special groups, including children and pregnant women.

## 1. Introduction

Systemic lupus erythematosus (SLE) is recognized as a chronic autoimmune disorder defined by inflammation and tissue damage across multiple organ systems. It affects an estimated 3.4 million individuals worldwide [1,2]. From a pathological perspective, the proliferative types—specifically Class III and IV, sometimes combined with Class V—represent the most aggressive forms of the disease. Advances in the understanding of LN pathogenesis have led to the development of novel therapies, allowing us to move away from high-dose glucocorticoids and broad-spectrum cytotoxic agents to more precise, multi-targeting strategies. This new paradigm is possible through the development and integration of biologic agents and validation of multi-targeted therapy that allow the minimization of glucocorticoids use. Moreover, potentially curative cell-based therapies are being developed. This evolution promises to improve clinical outcomes, but it will also introduce new complexities in clinical decision-making.

A comprehensive literature search was conducted using PubMed, Scopus, and Google Scholar databases with no restriction on publication date. Search terms included combinations of “lupus nephritis,” “treatment,” “therapy,” “pathogenesis,” “clinical trial,” and using specific drug names. Relevant clinical practice guidelines from the Kidney Disease: Improving Global Outcomes (KDIGO), the European Alliance of Associations for Rheumatology (EULAR), the American College of Rheumatology (ACR), and the U.S. Food and Drug Administration (FDA) were also reviewed. The list of references of identified articles were manually screened for additional relevant publications.

## 2. Pathogenesis of Lupus Nephritis

Understanding the pathogenesis of lupus nephritis (LN) requires navigating a multifaceted landscape. It is not triggered by a single event but results from a complex interplay whereby genetic predisposition collides with environmental factors, ultimately leading to a systemic breakdown in both innate and adaptive immune regulation [3]. Here, we focus on three highly interconnected pathways central to this process and relevant to current targeted therapies: the amplification of autoimmunity by type I interferon system, and subsequent B-cell and T-cell hyperactivity.

### 2.1. Interferon System

The excessive release of DNA- and RNA-protein complexes from dying cells drives the loss of tolerance to nuclear self-antigens. In patients with SLE, these nucleic acid complexes stimulate resident renal cells and infiltrating immune cells to synthesize type I interferon (IFN-I) [4]. IFN-I is highly pathogenic. It promotes the formation of immune complexes and recruits leukocytes that drive proliferative lesions, directly injuring resident renal cells that ultimately lead to apoptosis and fibrosis [5]. Moreover, this local IFN-I production within the kidney fosters a hostile inflammatory environment in the glomeruli, leading to renal fibrosis and nephron loss.

### 2.2. B-Cells

The nucleic acid complexes activate dendritic cells, monocytes, and macrophages through Toll-like receptors (TLRs), which then secrete pro-inflammatory mediators that activate B- and T-cells [6]. When an imbalance of B-cell maturation occurs, a persistent population of autoreactive B-cells is allowed to thrive [7,8]. These B-cells play a dual role: they not only generate autoantibodies but also serve as antigen-presenting cells further promoting T-cell activation and inflammation in LN.

Multiple cytokines are involved in B-cell development and differentiation. Among them, two specific molecules stand out as critical survival signals: the B-lymphocyte stimulator (BAFF) and a proliferation-inducing ligand known as APRIL [9,10,11]. In clinical observations of proliferative LN, both factors are found to be overexpressed in kidney tissue [12]. They are crucial for sustaining the reservoir of autoreactive B-cells and plasma cells responsible for generating pathogenic autoantibodies.

Importantly, there is a subset of autoreactive B-cells and long-lived plasma cells that reside within tissue niches, such as the bone marrow and inflamed kidneys, where they are sheltered from anti-CD20 therapies that target circulating B-cells. This tissue-resident population serves as a constant source for the autoantibody pool, driving persistent disease activity even after apparent peripheral B-cell depletion [13,14]. The persistence of these cells provides the immunological basis for deeper depletion strategies, such as anti-CD19 chimeric antigen receptor (CAR) T-cell therapy, which will be discussed in Section 6.

### 2.3. T-Cells

In LN, apart from the recruitment of T-cells to the injured kidney, the T-cell subsets are also dysregulated. There appeared a “dual dysfunction”: an expansion of aggressive effector populations contrary to functional impairment of regulatory mechanisms.

Specifically, the effector arm—comprising both CD4+ helper and CD8+ cytotoxic subsets—becomes hyperactive. The CD4+ lineage drives injury through two distinct vectors: first, by directly secreting a storm of pro-inflammatory cytokines (such as IL-17 and IFN-γ); and second, by providing the critical co-stimulatory signals that fuel B-cell hyperactivity and autoantibody synthesis [15]. Parallel to this, cytotoxic CD8+ cells migrate into the kidney tissue, leading directly to renal injury [16]. Under normal physiological conditions, regulatory T-cells (Tregs) serve as the guardians of immune tolerance. However, in the context of LN, their numbers and function are often impaired, contributing to a loss of tolerance and increased autoimmunity [17]. Central to this T-cell activation is the calcineurin/NFAT signaling pathway. Upon T-cell receptor engagement, calcineurin dephosphorylates NFAT transcription factors enable their nuclear translocation and transcription of pro-inflammatory cytokine genes—including IL-2, IL-4, and TNF-α—thereby amplifying T-cell-mediated glomerular inflammation and tubular injury [15,18]. This pathway represents a key therapeutic target for calcineurin inhibitors [19].

**Figure 1:**
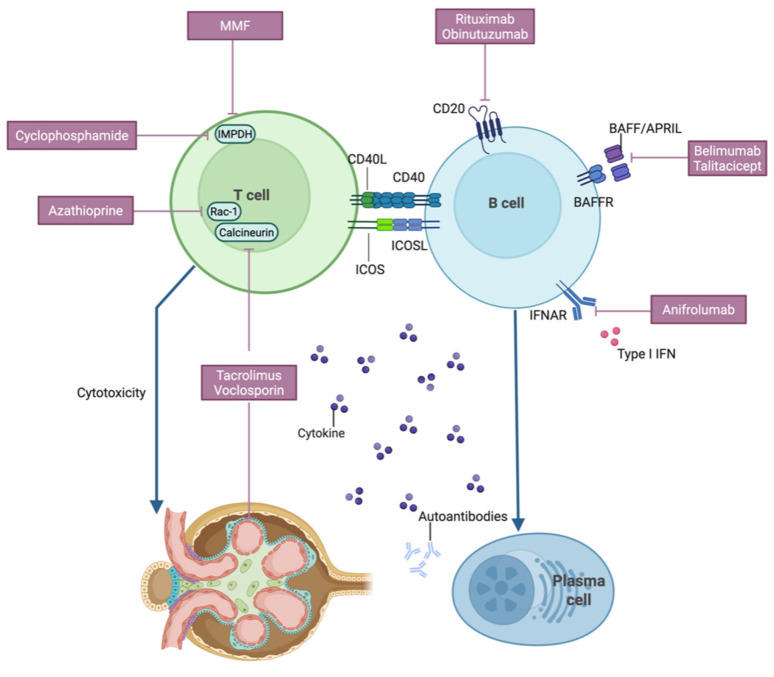
Key pathogenic pathways and the targets of various therapies in lupus nephritis. Activated T-cells directly injure the kidney through cytotoxicity and the release of inflammatory cytokines. T-cells also activate B-cells, which leads to the production of pathogenic autoantibodies by plasma cells. Therapies targeting the T-cell pathway include mycophenolate mofetil (MMF), cyclophosphamide, and azathioprine, which suppress T-cell proliferation, and calcineurin inhibitors like tacrolimus and voclosporin, which inhibit T-cell activation. B-cell targeted agents act either by depleting B-cells via the CD20 protein (rituximab, obinutuzumab) or by blocking key survival signals like BAFF and/or APRIL (belimumab, telitacicept). Additionally, the type I interferon pathway can be inhibited by anifrolumab, which blocks the IFNAR receptor. Created with BioRender.com.

The complex immunopathogenesis of LN has provided the basis for the development of new, targeted therapies. The key immune pathways driving renal injury, including the interplay of type I interferons, B-cells, and T-cells, are shown in Figure 1. Figure 1 also shows the sites of action of the major immunosuppressive and biologic agents being discussed in this review.

### 2.4. Histopathological Assessment and Its Role in Treatment Decisions

Renal biopsy remains the gold standard for the diagnosis and classification of LN. The International Society of Nephrology/Renal Pathology Society (ISN/RPS) classification categorizes LN into six classes based on the pattern of glomerular involvement. This histological classification determines the therapeutic decision-making. Proliferative forms (Class III and IV, with or without concurrent Class V) represent the most aggressive phenotypes and require intensive immunosuppressive induction therapy [20]. Beyond histological class, the National Institute of Health (NIH) activity index (AI) and chronicity index (CI) provide important prognostic information. A high AI indicates potentially reversible inflammation responsive to treatment intensification, whereas a high CI indicates irreversible scarring that may limit therapeutic benefit and predicts progression toward end-stage kidney disease [21,22].

Traditional clinical parameters often fail to accurately predict therapeutic response or early LN relapse [23]. Other than initial biopsy, repeat renal biopsy is proving to be a useful tool for the ongoing management of LN. Repeat biopsies performed after induction therapy have revealed that 29% of those in complete remission and 61% of those in partial remission, in fact, had active lesions on repeat biopsies [24]. Moreover, findings from the second biopsy, rather than the initial biopsy, have been shown to be stronger predictors of progression to end-stage kidney disease [25]. The chronicity index at repeat biopsy, in particular, has emerged as a strong independent predictor of long-term renal function loss [26,27]. These findings support the incorporation of repeat biopsy into a histology-guided treatment strategy for LN, where therapy is adjusted to achieve histological response rather than pure clinical response [28]. Despite its diagnostic value, the invasive nature of kidney biopsy has restricted its repeated use in clinical practice. This has driven the search for non-invasive alternatives such as transforming growth factor beta1, pentraxin-3, soluble CD163, CD11b, and interleukin-16; all of these show promise in mirroring the histopathological state of the kidneys [29].

## 3. A History of Therapeutic Evolution

### 3.1. The Use of Glucocorticoids

For decades, glucocorticoids have stood as the cornerstone of LN management due to their rapid and potent capacity to quell inflammation [30]. However, this efficacy comes at a high price. The reliance on high-dose, long-term steroid regimens imposes a heavy toxicity burden on patients. The cumulative side effects are extensive, ranging from metabolic complications like hyperglycemia and osteoporosis to an elevated risk of life-threatening infections and cardiovascular disease [31]. This treatment-related toxicity has driven the search for steroid-sparing strategies.

### 3.2. The Advent of Cytotoxic Agents and Mycophenolate Mofetil

Cyclophosphamide (CYC), an alkylating agent capable of broad lymphocyte and neutrophil suppression, marked a pivotal turn in LN therapy [32].

Landmark clinical trials spearheaded by the NIH during the 1970s and 80s cemented the role of CYC. These studies demonstrated that the combined cytotoxic agent with steroids significantly improved the long-term prognosis for renal function preservation compared to steroids alone [33,34,35]. However, the toxicity burden of high-dose CYC regimens prompted a search for safer alternatives. The Euro-Lupus Nephritis Trial introduced a low-dose intravenous (iv) CYC regimen that showed comparable efficacy to the high-dose NIH standard but offered a vastly superior safety profile, particularly within Caucasian cohorts [36].

Azathioprine (AZA), a purine analog that reduces B- and T-cell activity, inhibits B- and T-cell proliferation and dampens autoantibody production [32]. While initially used as a steroid-sparing agent, early cohort studies of patients with severe renal or CNS involvement suggested that AZA could sustain long-term survival outcomes similar to pulse CYC [37,38]. Later, several studies have shown that maintenance treatment with low-dose iv CYC and AZA were as effective as the high-dose CYC regimen but with fewer side effects [36,39,40]. This established AZA as a standard maintenance therapy for LN.

The quest for even safer alternatives led to the introduction of mycophenolate mofetil (MMF) in the 1990s. By selectively blocking de novo purine synthesis, MMF targets proliferating B- and T-cells with greater precision [32]. Several studies [41,42,43], including the pivotal Aspreva Lupus Management Study (ALMS), confirmed that the non-inferiority of MMF compared to intravenous CYC in inducing remission while offering a significantly reduced risk of infection [44,45]. Furthermore, a meta-analysis demonstrated that MMF was as effective as AZA as maintenance treatment [45]. Due to its comparable efficacy and more favorable safety profile, MMF has been widely used in both induction and maintenance therapy for LN. Common side effects of MMF include gastrointestinal symptoms such as diarrhea and nausea. But the more serious side effects, including leukopenia and serious infections, had led to MMF discontinuation in a significant proportion of patients [41]. Therefore, the search for more specific targeted and less toxic therapies remains a crucial objective.

### 3.3. The Evolution: A New Era of Targeted Treatment

#### 3.3.1. Belimumab

Belimumab is a human monoclonal antibody that inhibits BLyS, a cytokine essential for B-cell survival and differentiation. By targeting BLyS, belimumab reduces autoreactive B-cell populations and suppresses autoimmune response [46].

The efficacy of belimumab was established through the landmark BLISS-LN trial, a 2-year Phase III study of 448 active LN patients [47]. The data revealed that adding intravenous belimumab into standard regimens (MMF/AZA or CYC/AZA) significantly boosted renal outcomes: the primary efficacy renal response (PERR) at year 2 rose from 32% to 43%, with a corresponding increase in complete renal response (CRR) rates (30% vs. 20%). Furthermore, the belimumab treatment decreased the risk of renal-related events or death by nearly half (hazard ratio 0.51). Based on this evidence, updated guidelines from both KDIGO (2024) have endorsed belimumab as the first-line “add-on” strategy for LN. Subsequent real-world evidence has also supported its effectiveness in clinical practice [46].

#### 3.3.2. Tacrolimus

Tacrolimus is a calcineurin inhibitor (CNI) with dual-action approach. It does not only suppress T-cell activation by inhibiting calcineurin, but it also functions as a direct stabilizer of the podocyte cytoskeleton, thereby reducing proteinuria [19]. The concept of multi-targeted therapy involves the simultaneous use of multiple agents with different mechanisms of action. This strategy aims to achieve synergistic efficacy while minimizing toxicity using lower drug dosages [48]. A multicenter, randomized trial has evaluated a multi-targeting induction regimen of tacrolimus and MMF against standard iv CYC in Chinese population. Both groups received initial methylprednisolone pulse therapy, followed by oral prednisone. The multi-targeted regimen was superior to iv CYC, with a significantly higher CRR at 24 weeks (45.9% vs. 25.6%). This multi-targeted regimen also showed fewer adverse events compared to an azathioprine-based regimen in a subsequent maintenance study [49]. However, the high-level evidence for this approach has derived predominantly from Asian populations. Therefore, its generalizability to other ethnic groups requires further investigation [50].

#### 3.3.3. Voclosporin

As a next-generation CNI, voclosporin distinguishes itself through a structurally modified profile that eliminates the need for the therapeutic drug monitoring [51]. Its efficacy was demonstrated in the AURORA 1 trial, where the addition of voclosporin to a therapy of MMF and steroids nearly doubled the complete renal response rate at 52 weeks (41% vs. 23% *p* < 0.001) [52]. Long-term follow-up from the AURORA 2 extension study confirmed these benefits over a 3-year period. The voclosporin group showed significantly greater preservation of eGFR and sustained reduction in proteinuria, leading to a higher CRR at 3 years (50.9% vs. 39.0%). The long-term safety profile was also favorable [53]. Based on this evidence, a triple-therapy regimen of voclosporin, MMF, and glucocorticoids is recommended as a first-line treatment by the KDIGO 2024 guideline, especially for patients with nephrotic-range proteinuria [20,54].

#### 3.3.4. Rituximab

Rituximab, a chimeric anti-CD20 antibody, effectively depletes circulating B-cells, but it showed variable results in clinical testing [55]. The LUNAR trial failed to meet its primary endpoint, showing no statistically significant difference in renal response rates at 52 weeks after adding rituximab to MMF plus glucocorticoids’ therapy (56.9% vs. 45.8%; *p* = 0.18), despite improvements in serologic markers. However, the rigidity of the trial design may have masked its potential utility [56]. Real-world observational data consistently highlights its value in refractory LN [57]. Consequently, rituximab remains in clinical use for this indication and is included as a therapeutic option in some clinical guidelines [58].

#### 3.3.5. Obinutuzumab

Obinutuzumab is the next-generation, humanized anti-CD20 monoclonal antibody, engineered for more profound B-cell depletion than its predecessor, rituximab. This theoretical advantage translated into clinical success in the Phase III REGENCY trial. Unlike the LUNAR study, REGENCY met its primary endpoint, demonstrating a statistically superior complete renal response rate at 76 weeks compared to placebo (46.4% vs. 33.1%; *p* = 0.02). Beyond efficacy, it offered a meaningful steroid-sparing effect without increasing the incidence of severe infections [59]. These findings have been supported by multiple network meta-analyses, which consistently rank obinutuzumab among the most effective biologics for achieving renal remission in LN. These analyses suggest efficacy superior torituximab and comparable to, or better than, belimumab and voclosporin [60,61,62]. Following the FDA approval of obinutuzumab for the treatment of adults with LN, obinutuzumab is poised to join belimumab and voclosporin as a top-tier therapeutic option.

#### 3.3.6. Anifrolumab

Identifying that type I interferon (IFN) pathway is a key driver of SLE inflammation has led to the study of anifrolumab, a monoclonal antibody blocking the IFN receptor subunit 1 (IFNAR1) in LN [63,64]. However, the Phase II TULIP-LN trial that evaluated anifrolumab as an initial treatment for proliferative LN, did not meet its primary endpoint of changes in proteinuria at 52 weeks [65]. This result was potentially attributable to the insufficient drug exposure in patients with severe proteinuria [66]. The data from a 2-year extension study has suggested a potential benefit with higher dose regimen, which informed the design of the ongoing Phase III IRIS trial [67].

Anifrolumab is currently approved for non-renal SLE. Its role in the treatment of LN will be clarified by the results of Phase III trials. It represents a potential strategy for biomarker-guided therapy, particularly for patients with a high IFN gene signature.

#### 3.3.7. Talitacicept

Telitacicept has emerged as a novel TACI-Fc fusion protein. It neutralizes both BLyS and APRIL, offering a dual-blockade strategy [68]. The potential of telitacicept was underscored by a pivotal Phase III trial involving 335 Chinese SLE patients. In this study, the addition of telitacicept (160 mg) to standard-of-care yielded an impressive SRI-4 response rate of 82.6% vs. placebo group (38.1%) at week 52 [69]. Furthermore, telitacicept has also demonstrated potential renal benefits in a 52-week real-world study of 51 Chinese LN patients, with 73.3% achieving PERR and 68.4% achieving CRR at the end of 1 year [70].

Based on this promising evidence, formal prospective trials are underway to evaluate telitaciceptin LN. Among the most important is the Phase 2, randomized, double-blind, placebo-controlled study of telitacicept in patients with lupus nephritis (NCT05680480), which is currently recruiting participants. The next step for establishing the definitive role of telitacicept in LN requires global multicenter randomized controlled studies that include ethnically diverse populations.

**Figure 2:**
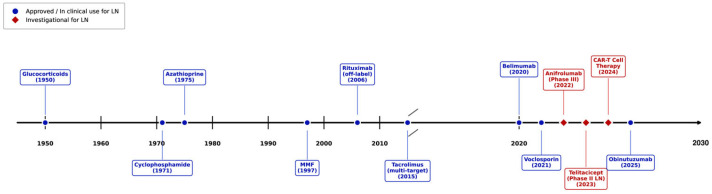
Timeline of therapeutic agents for lupus nephritis. Blue circles represent agents approved or established in clinical practice for LN. Red diamonds indicate investigational agents currently in clinical trials. The timeline uses a non-linear scale in order to allow for the rapid drug development in the last decade. Created with matplotlib (v3.7.2).

The timeline of key therapeutic agents for LN is illustrated in Figure 2. A summary of the mechanism, efficacy, safety, and recommendations for each agent is presented in Table 1.

## 4. A New Standard-of-Care: Combination Therapy

The success of these new, targeted agents has prompted a fundamental shift in the management of proliferative LN. Current international guidelines from organizations such as KDIGO and EULAR now recommend initiating treatment with combination therapy and tapering the glucocorticoids rapidly.

A rapid glucocorticoid tapering schedule is essential for reducing cumulative toxicity. The protocol used in the AURORA trials enabled the tapering of prednisone to 2.5 mg/day by week 16, with favorable safety profile [52]. The long-term objective is to reduce the maintenance prednisone dose to ≤5 mg/day, with the aim of complete withdrawal in those patients who achieve sustained remission [74].

### Current KDIGO Guideline Recommendations

For active proliferative LN, the 2024 KDIGO guideline recommends initial therapy with glucocorticoids combined with one of four regimens: mycophenolate mofetil (MMF), cyclophosphamide (CYC), belimumab, or calcineurin inhibitor (CNI), e.g., voclosporin [20]. The choice is tailored to the patient’s clinical profile. For instance, because of the direct podocyte-stabilizing effects of CNI, CNI-based regimens are usually favored in patients with heavy, nephrotic-range proteinuria [20]. In LN patients with significant extra-renal activity or with history of frequent relapses, a belimumab-based strategy may offer better systemic control [20]. Ethnicity also influences therapeutic choices. MMF is preferred to CYC as an induction agent for patients of African and Hispanic ancestry [75,76,77].

Following successful induction therapy, it is recommended that patients should receive maintenance therapy for at least 3 years to prevent relapse and preserve renal function. It is also recommended that the prednisone dose be reduced to a low level of maintenance dose (≤5 mg/day) over 3–6 months. According to the 2024 KDIGO guideline, MMF is the first-line agent for maintenance in most patients. Alternatives include the use of a CNI-based triple therapy if CNI has been used for induction or AZA if MMF is unavailable or intolerable [20].

## 5. Special Considerations

### 5.1. Refractory Disease

In patients who fail to respond to standard therapy, it is important to assess for treatment adherence [78]. A repeat renal biopsy may be necessary if there is a failure to achieve therapeutic response or in cases of renal flare. Therapeutic strategies in refractory diseases include:(1) switching the primary immunosuppressive agent (e.g., from MMF to CYC or vice versa) or (2) intensifying treatment by adding a CNI to MMF in accordance with a multi-targeted approach [77]. Rituximab may be considered as an off-label therapy in this situation [79,80]. Furthermore, in a real-world study [81] of nine patients with LN refractory to rituximab, a switch to obinutuzumab led to both clinical and biochemical improvements. These findings have made obinutuzumab a promising therapeutic option for refractory LN.

### 5.2. Children and Adolescents

LN is often more severe in children and adolescents. Managing LN in these special groups requires a delicate balance between aggressive disease control and the preservation of growth and fertility [82]. Glucocorticoids may impair growth, while CYC carries a significant risk of infertility. Hence, minimizing the use of glucocorticoids and CYC is a priority. CNIs are considered to be an effective therapeutic option for children. In addition, belimumab has been approved by the FDA, and it is another effective therapy for SLE in children over 5 years old. Rituximab may be used in children with refractory LN in combination with another disease-modifying antirheumatic drug (DMARD), including hydroxychloroquine, methotrexate, AZA, and MMF [83]. A structured transition from pediatric to adult care is also an important component for long-term management [82].

### 5.3. Lupus Nephritis and Pregnancy

LN may flare up during pregnancy, making its management more challenging. The planning for pregnancy requires careful preconception counseling and management [84]. Disease quiescence for at least 6 months prior to conception is the golden rule for minimizing risks [20]. During pregnancy, the use of glucocorticoids, hydroxychloroquine, azathioprine, and CNIs (tacrolimus and cyclosporine) are considered acceptable. In contrast, MMF and CYC are strictly forbidden due to their teratogenic potential. Patients on MMF must undergo a carefully timed “washout” and switch to azathioprine before attempting conception. Emerging data on belimumab suggests a potential safety profile [85]. Belimumab is labeled as a FDA-category C drug, and it is not recommended in pregnancy.

### 5.4. Racial and Ethnic Difference

The incidence, severity, and outcomes of LN are very variable between different racial and ethnic groups. The disease is more common and severe in patients of African, Hispanic, and Asian descent [86,87]. These disparities are due to multiple factors, including genetic factors (e.g., APOL1 risk variants of African descent) and socioeconomic determinants of health [88,89]. These factors also contribute to a higher risk of progression to ESKD [90]. Recognizing these disparities is essential in order to provide equitable care and effective treatment strategies. For example, MMF has been shown to be more effective than CYC as an induction therapy for African American and Hispanic patients [91,92], whereas azathioprine is considered a reasonable maintenance therapy for Hispanic populations [92]. In Asian patients, combination therapy using MMF and CNI is increasingly preferred for induction due to its superior remission rates [49,93]. In China, telitacicept has emerged as a new addition in the combination therapy regimen for SLE patients with or without LN.

## 6. The Future of LN Management

With the development of more targeted therapies, it is hoped that clinicians are able to provide more precise and personalized therapy to LN patients while minimizing adverse effects. In patients with refractory disease, the emergence of chimeric antigen receptor (CAR)-T-cell therapy may provide curative potential in the future. Unlike conventional anti-CD20 antibodies (rituximab, obinutuzumab) that primarily deplete circulating B-cells, anti-CD19 CAR-T-cells can access and eliminate B-cells within tissue niches that include tissue-resident memory B-cells and early plasmablasts in the bone marrow and inflamed organs, and it enables a more profound depletion of B-cells [13]. The elimination of this tissue-resident reservoir, as discussed in Section 2.2, is likely to explain the sustained remissions observed with CAR-T approaches. Early clinical studies have reported encouraging results. In a Phase 1 trial involving two patients with treatment-resistant Class IV LN, anti-CD19 CAR-T-cell therapy induced clinical improvement, and it was well-tolerated [71]. Similarly, cohort studies of patients with severe, refractory SLE (including patients with proliferative LN) have achieved high rates of rapid, drug-free remission, following deep B-cell depletion with the normalization of disease activity markers [72,73,94]. Following CAR-T-mediated deep depletion, the reconstituting B-cell repertoire appeared to reset toward a naive, non-autoreactive phenotype. This might explain the sustained drug-free remissions observed in these early studies. Ongoing CAR-T clinical trials are underway to determine the optimal dosing, long-term safety, and potentially extending this approach to less severe forms of LN.

## 7. Conclusions

This review has presented the evolution of immunotherapy for LN, and it has taken the audience through half a century of the drug development. The management of LN has shifted from a reliance on broad-spectrum immunosuppression toward specific and targeted therapies. Building on the basis of MMF or low-dose CYC, the addition of new targeted agents, such as belimumab or voclosporin, has optimized the clinical outcome while reducing overall toxicity. These new multi-targeting strategies have allowed rapid taper of steroid after initial pulse, thereby reducing long-term steroid toxicity. For refractory patients, more treatment options are available that target B-cells to plasma cells to APRIL and BLyS. We are now able to apply more personalized therapies according to patients’ ethnicity, age, and gender. However, more research is needed on how to best use these drugs, whether singly or in combination, and on how to determine their duration. With ongoing research, including CAR-T-cell therapy in LN, a about better and brighter future should emerge for patients with LN.

## Figures and Tables

**Table 1:** Summary of therapeutic agents for lupus nephritis: mechanism, efficacy, safety, and current recommendations.

Drug	Mechanism of Action	Key Trial/Efficacy	Adverse Effects	Recommendation
Glucocorticoids	Broad anti-inflammatory and immunosuppressive [30].	Foundational therapy; decades of clinical use.	Hyperglycemia, osteoporosis, infections, CVD [31].	Taper to ≤5 mg/d; aim for withdrawal [20].
Cyclophosphamide (CYC)	Alkylating agent; broad lymphocyte and neutrophil suppression [32].	NIH trials [33,34,35]; Euro-Lupus: comparable efficacy with lower toxicity [36].	Gonadal toxicity, infections, myelosuppression. *	Low-dose IV CYC for induction (KDIGO 2024) [20].
Azathioprine (AZA)	Purine analog; inhibits B- and T-cell proliferation [32].	Similar efficacy to CYC for maintenance [36,39,40]; comparable to MMF [45].	Myelosuppression, hepatotoxicity. *	Maintenance if MMF unavailable/intolerable [20].
Mycophenolate Mofetil (MMF)	Selective inhibitor of de novo purine synthesis [32].	ALMS: non-inferior to IV CYC for induction [44,45].	GI symptoms, leukopenia, serious infections. [41]	First-line induction and maintenance (KDIGO 2024) [20].
Belimumab	Anti-BLyS monoclonal antibody; reduces autoreactive B-cells [46].	BLISS-LN: CRR 30% vs. 20% at 2 yr; PERR 43% vs. 32% [47].	Infections, infusion reactions. *	First-line add-on (KDIGO 2024) [20].
Tacrolimus	CNI; suppresses T-cell activation, and acts as a podocyte stabilizer [19].	Multi-target trial: CRR 45.9% vs. 25.6% at 24 wk [48,49].	Nephrotoxicity, glucose intolerance, tremor. *	Multi-target induction; mainly Asia evidence [50].
Voclosporin	Next-generation CNI; no drug monitoring required [51].	AURORA 1: CRR 41% vs. 23% at 52 wk [52]; AURORA 2: sustained at 3 yr [53].	Hypertension, GFR decline, diarrhea. *	First-line triple therapy (KDIGO 2024) [20,54].
Rituximab	Chimeric anti-CD20 antibody; depletes circulating B-cells [55].	LUNAR: ORR 56.9% vs. 45.8% (*p* = 0.18, not sig.) [56].	Infusion reactions, infections, PML (rare). *	Off-label for refractory LN [58].
Obinutuzumab	Humanized anti-CD20; enhanced B-cell depletion vs. rituximab.	REGENCY: CRR 46.4% vs. 33.1% at 76 wk (*p* = 0.02) [59].	Infections, infusion reactions [59].	FDA-approved for LN [59].
Anifrolumab	Anti-IFNAR1; blocks type I IFN signaling [63,64].	TULIP-LN (Phase II): primary endpoint not met [65].	Herpes zoster, URI. *	Approved for non-renal SLE; Phase III IRIS ongoing [67].
Telitacicept	TACI-Fc fusion protein; dual BLyS/APRIL blockade [68].	Phase III SLE: SRI-4 82.6% [69]; LN real-world study: PERR 73.3%, CRR 68.4% [70].	Infections, injection site reactions. *	NMPA-approved for SLE; Phase 2 RCT in LN ongoing (NCT05680480).
CAR-T-Cell therapy	Anti-CD19 CAR-T; deep B-cell depletion, including tissue niches.	Phase 1: clinical improvement in Class IV LN [71]; drug-free remission [72,73].	CRS, neurotoxicity, infections. *	Experimental; refractory SLE/LN.

Abbreviations: CRR, complete renal response; PERR, primary efficacy renal response; ORR, overall renal response; CNI, calcineurin inhibitor; BLyS, B-lymphocyte stimulator; APRIL, a proliferation-inducing ligand; CRS, cytokine release syndrome; CVD, cardiovascular disease; GI, gastrointestinal; PML, progressive multifocal leukoencephalopathy; URI, upper respiratory infection. * Adverse effects compiled from the respective product labels and pivotal clinical trials.

## Data Availability

No new data were created.
